# Respiratory Muscle Injury Following Acute Monocled Cobra (*Naja kaouthia*) Envenoming: Histopathological Study in Rat Diaphragm

**DOI:** 10.3390/cimb47020086

**Published:** 2025-01-29

**Authors:** Wanida Chuaikhongthong, Wipapan Khimmaktong, Natyamee Thipthong, Nissara Lorthong, Janeyuth Chaisakul

**Affiliations:** 1 Division of Health and Applied Sciences, Faculty of Science, Prince of Songkla University, Songkhla 90110, Thailand; 6510220030@email.psu.ac.th (W.C.); wipapan.k@psu.ac.th (W.K.); 6510220006@email.psu.ac.th (N.T.); 6410230018@email.psu.ac.th (N.L.); 2 Department of Pharmacology, Phramongkutklao College of Medicine, Bangkok 10400, Thailand

**Keywords:** diaphragm, myotoxicity, antivenom, snakebite, histology

## Abstract

Clinical symptoms of monocled cobra (*Naja kaouthia*) envenoming include the paralysis of extraocular muscles, local tissue necrosis and death through respiratory failure. These neurotoxic outcomes are mainly due to the inhibitory action of postsynaptic neurotoxins to nicotinic acetylcholine receptors. However, injuries involving respiratory muscles have rarely been investigated. In this study, we determined the effect of *N. kaouthia* envenoming on morphological changes in the rat diaphragm. The efficacy of cobra monovalent antivenom in neutralising the histopathological effects of *N. kaouthia* venom was also evaluated. The intramuscular (i.m.) administration of *N. kaouthia* venom (2 mg/kg) caused skeletal muscle fibre atrophy and ruptures of myofibrils shown via a light microscope study. Transmission electron microscopy (TEM) revealed the zig-zagging of the Z-band, mitochondrial damages and degeneration of the synaptic fold of the neuromuscular junction following experimental cobra envenoming for 4 h. Intravenous administration of cobra antivenom at manufacturer-recommended doses diminished histopathological changes in the diaphragm following the administration of cobra venom. The expression of NF-kB and MuRF1 in the experimentally *N. kaouthia-*envenomed diaphragm indicated inflammation and tissue atrophy in the immunofluorescence analysis, respectively. In this study, we found that there were respiratory muscle injuries following *N. kaouthia* envenoming. The early administration of monovalent *N. kaouthia* antivenom is capable of neutralising neurotoxic outcomes following cobra envenoming.

## 1. Introduction

Cobras (*Naja* sp.) are medically important snakes in Africa and Asia. Four species of the genus *Naja* are found in Thailand: *Naja siamensis* (the Indochinese spitting cobra), *Naja kaouthia* (the monocled cobra), *Naja sumatrana* (the Equatorial splitting cobra) [1,2] and the newly identified species *Naja fuxi* [3]. In 2010, the World Health Organization classified *N. siamensis* and *N. kaouthia* as category 1 medically important venomous species that cause high-level disability and mortality in the Thai population [4].

In Thailand, monocled cobras are commonly found in the central and southeastern regions and are also seen in the north and northeast [2]. Neurotoxicity, including ptosis, ophthalmoplegia and progressive neuromuscular paralysis leading to respiratory failure, is the most significant outcome following *N. kaouthia* envenoming [5,6,7]. Moreover, local tissue necrosis, swelling and cardiovascular disturbances (i.e., hypertension and tachycardia) are also observed [6]. Mostly, tissue necrosis is seen between 80 and 120 min following cobra envenoming. Surgical wound care and antibiotics are required to prevent and control severe infection [6,7,8].

Early antivenom administration and respiratory support (i.e., endotracheal intubation with ventilator support) are essential for the management of systemic cobra envenoming. The Queen Saovabha Memorial Institute (Thai Red Cross Society, Bangkok, Thailand) is the only manufacturer of *N. kaouthia* antivenom (NKAV) in Thailand. It also produces the Neuro Polyvalent Snake Antivenom for Southeast Asian elapid envenoming, which covers the venoms of *Ophiophagus hannah*, *N. kaouthia*, *Bungarus fasciatus* and *Bungarus candidus* [9].

Neurotoxicity observed following envenoming by cobras is attributed to the presence of pre- and postsynaptic neurotoxins [10]. Presynaptic neurotoxins interrupt the cholinergic transmitter (i.e., acetylcholine [ACh]) release, synthesis, storage or turnover in the synaptic nerve terminal [11], while postsynaptic neurotoxins inhibit the interaction of ACh with the skeletal muscle subtype of the nicotinic ACh receptor [12]. A proteome analysis showed that phospholipase A_2_ [PLA_2_] and three-finger toxins (3FTxs) were the most abundant protein families detected in *N. kaouthia* venom [13,14]. Other minor protein families (i.e., L-amino acid oxidase and kunitz-type serine inhibitors), including some unique proteins such as waprin, vespyrn, nerve growth factor and metalloproteinase, were also detected [13].

Although neuromuscular disturbance involving respiratory failure appears to be the most significant life-threatening phenomenon observed in cobra-envenomed patients, the pathological involvement of respiratory muscles behind this outcome has not been fully investigated. Further understanding and investigation of the pathology of the associated respiratory muscle could have significant benefits in improving the management of severe cobra envenoming in these regions. This study aimed to determine the histopathological changes of the diaphragm and neuromuscular junction following the administration of *N. kaouthia* venom in an anaesthetised rat model. The efficacy of NKAV to minimise the evidence of morphological changes in experimentally cobra-envenomed rat diaphragms was also investigated. Moreover, immunohistochemical analyses were performed to determine the expression of inflammatory proteins in skeletal muscle.

## 2. Materials and Methods

### 2.1. Animal Ethics and Care

All methods were performed in accordance with the relevant guidelines and regulations (https://arriveguidelines.org, accessed on 7 January 2024). In brief, male Sprague–Dawley rats were purchased from Nomura-Siam International Co. Ltd., Bangkok, Thailand. All animals were maintained on a regular diurnal lighting cycle (12:12 light–dark). Two or four rats were housed together in individual stainless-steel cages with ad libitum access to food and drinking water. Chopped corn cobs were used as bedding. Approval for all experimental procedures was obtained from the Subcommittee for Multidisciplinary Laboratory and Animal Usage of Phramongkutklao College of Medicine and the Institutional Review Board, Royal Thai Army Department, Bangkok, Thailand (documentary proof of ethical clearance number: IRBRTA S029b/67_Xmp on 9 April 2024) in accordance with the 1986 U.K. Animal (Scientific Procedure) Act and the National Institutes of Health’s Guide for the Care and Use of Laboratory Animals (NIH Publications 8th edition, 2011).

### 2.2. Snake Venoms

Pooled monocled cobra venom (*N. kaouthia*) was a gift from Professor Nison Sattayasai (Department of Biochemistry, Faculty of Science, Khon Kaen University, Thailand). The venom sample was obtained by milking from several specimens held captive in a snake farm at the Queen Saovabha Memorial Institute (QSMI) of the Thai Red Cross Society in Bangkok, Thailand. Freeze-dried venom samples were stored at 4 °C prior to use. When required, the venom was weighed and reconstituted in phosphate-buffered saline (PBS), and the protein concentration was measured using a BCA protein assay (Pierce Biotechnology, Rockford, IL, USA).

### 2.3. Antivenoms

The monocled cobra (*N. kaouthia*) monovalent antivenom (Lot no. NK00321; expiry date: 20 August 2026) was purchased from QSMI. The freeze-dried antivenoms were dissolved with pharmaceutical-grade water supplied by the manufacturer. The dissolved antivenoms were then stored at 4 °C prior to use.

### 2.4. Anaesthetised Rat Preparation

To monitor blood pressure and heart rate during the entire experiment, male Sprague–Dawley rats weighing 280–350 g (*n* = 3–5 per group) were anaesthetised using separate injections of Zoletil (20 mg/kg, i.p., Virbac, TX, USA) and Xylazine (5 mg/kg, i.p., Kepro, Woerden, The Netherlands). Additional anaesthetic was administered throughout the experiment as required. A midline incision was made in the cervical region, and cannulae were inserted into the trachea, jugular vein and carotid artery for artificial respiration (if required), the administration of antivenom and the measurement of blood pressure, respectively. Arterial blood pressure was recorded using a Gould Statham P23 pressure transducer filled with heparinised saline (25 U/mL). Systemic blood pressure was monitored using a MacLab system (ADInstruments, Bella Vista, Australia). Pulse pressure was defined as the difference between systolic and diastolic blood pressure. Mean arterial pressure was defined as diastolic blood pressure plus one-third of pulse pressure. The rats were kept under a heat lamp for the entire experiment to maintain body temperature. The i.m. administration of *N. kaouthia* venom was conducted through the right gastrocnemius muscle.

#### 2.4.1. Preliminary Experiment to Determine Venom Dose

Preliminary experiments examined the effects of *N. kaouthia* venom, administered to three rats, at doses of 0.5, 1.0 and 2.0 mg/kg. Venom doses of 0.5 and 1.0 mg/kg (i.m.) did not cause morphological changes of diaphragms. Subsequently, a dose of 2 mg/kg (i.m.) was chosen for further experiments.

#### 2.4.2. Determination of *N. kaouthia* Monovalent Antivenom Effectiveness

Where indicated, monovalent *N. kaouthia* antivenom (NKAV, Lot no. NK00321; expiry date: 20 August 2026) at one time (i.e., 1.0 mL for 300 g rat) the venom/antivenom ratio of the recommended therapeutic dose (i.e., 1 mL antivenom per 0.6 mg *N. kaouthia* venom) was manually administered via the jugular vein at an infusion rate of 0.25 mL/min over 3–4 min. Antivenom was administered 30, 60 and 90 min after venom administration.

### 2.5. Histological Preparation

A histopathological examination of the diaphragm of envenomed rats was carried out following previously described methods [15].

#### 2.5.1. Haematoxylin and Eosin (H&E) Staining

At the conclusion of the in vivo tests (i.e., 4 h), the surviving rats were euthanised, and their diaphragms were removed, washed with PBS and preserved in 10% formalin for light microscope and transmission electron microscopy (TEM) studies. All tissues were dehydrated in a graded series of ethanol at 70%, 80%, 90%, 95% and 100%, with two changes for 1 h each. Three washings of xylene for 30 min each were then performed before embedding the tissues in paraffin. The embedded samples were cut into cross-sections and stained with H&E. The tissues were examined and photographed using an Olympus light microscope (BX-50, Olympus, Tokyo, Japan).

#### 2.5.2. Transmission Electron Microscopy (TEM)

Pieces of diaphragm tissues (~1 mm^3^) were immediately fixed in 2.5% buffered glutaraldehyde. The specimens were post-fixed in 1% osmium tetroxide, dehydrated, infiltrated with propylene oxide and embedded in resin. Semi-thin sections of approximately 0.5 µm or 1.0 µm were stained with Toluidine blue, used as a guideline to the area of interest and further trimmed. Ultrathin sections of about 60 nm were cut on an ultramicrotome and then stained with uranyl acetate and lead citrate. The ultrathin sections were spread mostly on 200 or 300 mesh copper grids and stained with uranyl acetate and lead citrate solutions. The sections were then examined and photographed using a transmission electron microscope (TEM-JEM2010, JEOL, Ltd., Tokyo, Japan). The criteria used to score the morphological changes of the rat diaphragms are described in Table 1.

#### 2.5.3. Immunofluorescence Analysis

The diaphragm tissue was separated and fixed in 10% formalin for 1 week. On the first day, the diaphragm tissue was washed in xylene twice for 10 min and hydrated in a graded series of ethanol at 100%, 90%, 70% and 50% for 20 min and blocking serum for 1 h in 2% Horse serum–0.3% Triton x-100–0.1 M PBS. The tissue samples were then incubated overnight at +4 °C with either NF-kB antibody (AB16502, Abcam, Cambridge, UK; dilution: 1:200) or MuRF1 (AB201941, Abcam, Cambridge, UK; dilution: 1:200). On the second day, secondary antibodies were applied for 2 h at room temperature after the diaphragm samples were washed three times in 0.1 M PBS. VectaFluor was used as the secondary antibody. The diaphragm samples were mounted with Vectasheild after final washing.

Images were examined and photographed under a fluorescence microscope (Olympus D73 with CellSens software (version 1.16). The NF-kB/MuRF1 percentage of cell expression was determined using ImageJ software (version 1.54g) to measure the fluorescence intensity. Diaphragm muscles were randomly selected from each specimen. The OD was normalised to measure the level of immunostaining in the muscle fibre area. The OD was determined as OD = ID − (M × MGV), where ID is the integrated density of the selected muscle fibre, M is the area of the selected muscle fibre region and MGV is the mean grey value of the background readings.

### 2.6. Data Analysis and Statistics

For the anaesthetised rat experiments, the sample size was based on the number of animals required to provide >85% power to detect an effect size of 35% with a confidence level (α) of 5% for the in vivo endpoint measure of blood pressure (standard deviation [SD] < 15%). This ensured that the experimental design was sufficiently powered. The statistical analysis was performed using Prism 9.4.0 software (GraphPad Software, San Diego, CA, USA). Multiple comparisons were made using ANOVA, followed by Tukey’s multiple comparison test. Values of *p* < 0.05 were accepted as significant. Data were expressed as the mean ± SD.

## 3. Results

### 3.1. Effect of Intramuscular (i.m.) Administration of N. kaouthia Venom on Anaesthetised Rats

The i.m. administration of *N. kaouthia* venom at doses of 2 mg/kg (4xLD_50_: *n* = 3) caused cardiovascular collapse and the absence of heart rate within 4 h.

### 3.2. Histopathological Changes in Rat Diaphragm Following i.m. Administration of N. kaouthia Venom

**Figure 1:**
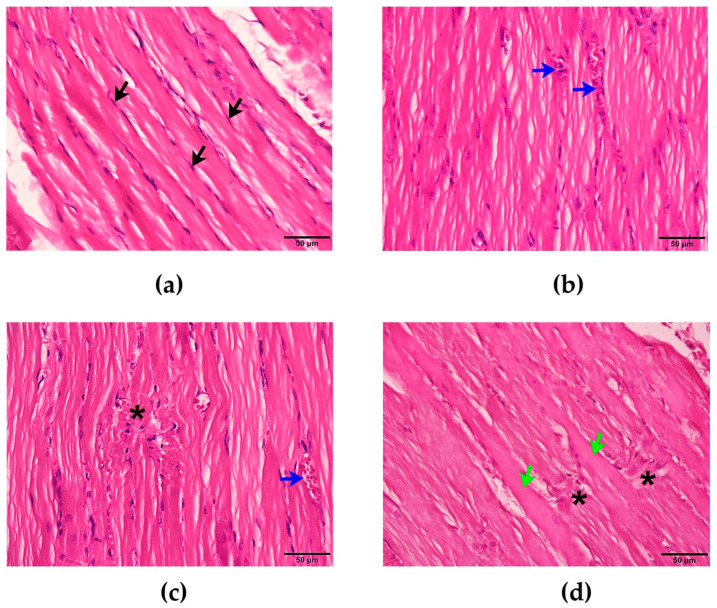
(**a**) Morphological changes of rat diaphragm after i.m. administration of saline (200 µL) showing the nucleus of the skeletal muscle cell (black arrows: H&E staining: scale bar = 50 µm). Morphological changes of rat diaphragm following administration of *N. kaouthia* venom (2 mg/kg) for (**b**) 60 and (**c**) 90 min showing red blood cell aggregations (blue arrow: H&E staining; scale bar = 50 µm) and myofibril ruptures (black asterisk: H&E staining; scale bar = 50 µm), respectively. (**d**) Morphological changes of rat diaphragm following the administration of *N. kaouthia* venom (2 mg/kg) for 4 h (H&E staining; scale bar = 50 µm: black asterisks indicate myofibril ruptures, green arrows indicate muscle hypertrophy).

**Figure 2:**
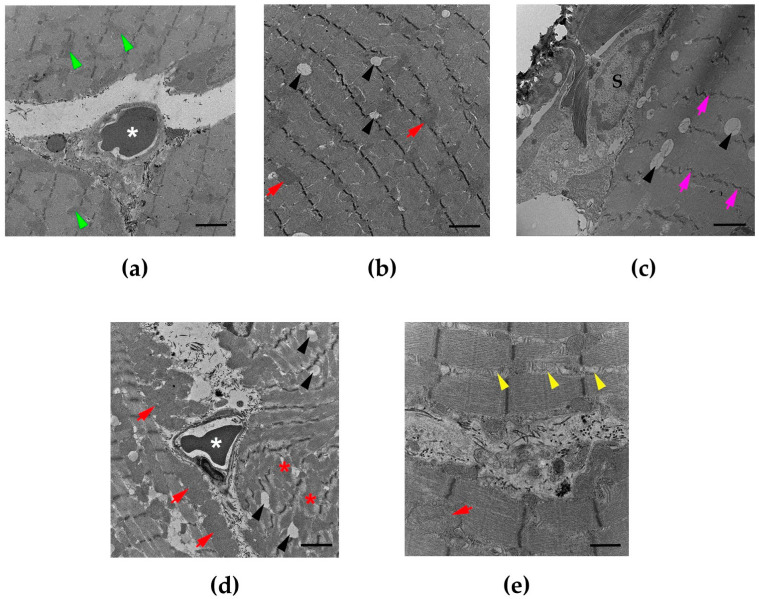
(**a**) Transmission electron microscopy (TEM) image of diaphragm following the administration of saline shows the mitochondria (green arrowheads) and the red blood cells (white asterisk: scale bar = 2 µm). Morphological changes of rat diaphragm under TEM showing (**b**) mitochondrial swelling (red arrows: scale bar = 2 µm), intramyocellular lipid droplets (black arrowhead: scale bar = 2 µm) and (**c**) the zig-zagging of the Z-band (pink arrows: scale bar = 2 µm) following the administration of *N. kaouthia* venom (2 mg/kg, i.m.) for 90 min. (**d**) TEM image of rat diaphragm following administration of *N. kaouthia* venom (2 mg/kg) for 4 h showing a high degree of muscle fibre degeneration (red asterisks), mitochondrial swelling (red arrows), red blood cells (white asterisk) and intramyocellular lipid droplets (black arrowheads: scale bar = 2 µm). (**e**) TEM image of rat diaphragm showing mitochondrial degeneration (yellow arrowheads: scale bar = 2 µm) following the administration of *N. kaouthia* venom (2 mg/kg, i.m.) for 4 h. ‘S’ indicates the nucleus of satellite cells.

**Figure 3:**
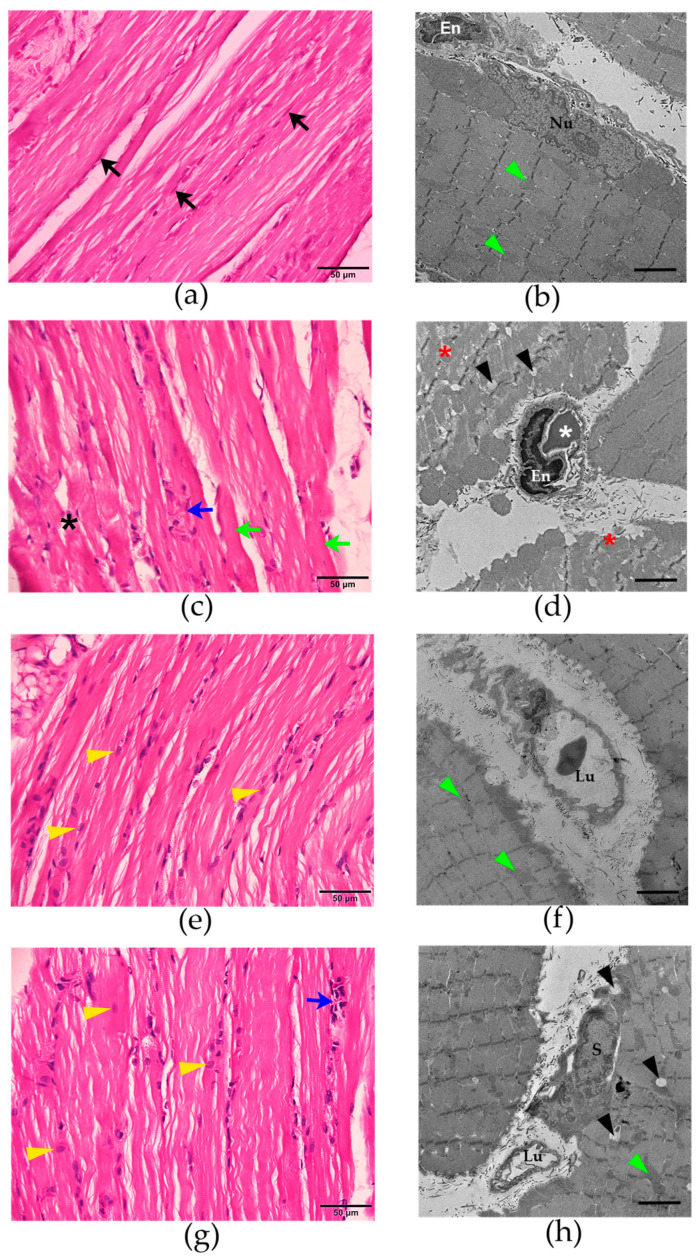
Morphological changes of the rat diaphragm after the i.m. administration of (**a**) saline (200 µL) showing the nucleus of the skeletal muscle cell (black arrows; H&E staining; scale bar = 50 µm) and (**b**) the mitochondria (green arrowheads; TEM image; scale bar = 2 µm). (**c**) Morphological changes of rat diaphragm (H&E staining; scale bar = 50 µm) following the administration of *N. kaouthia* venom (2 mg/kg: i.m.) for 4 h showing myofibril ruptures (black asterisks), red blood cell aggregations (blue arrow) and skeletal muscle hypertrophy (green arrows). (**d**) TEM image of rat diaphragm (scale bar = 2 µm) following the administration of *N. kaouthia* venom (2 mg/kg: i.m.) for 4 h shows high degree of muscle fibre degeneration (red asterisks), endothelial cell swelling (En), the appearances of red blood cells (white asterisk) and intramyocellular lipid droplets (black arrowheads). (**e**) H&E staining image and (**f**) TEM analysis (scale bar = 2 µm) of the rat diaphragm after i.v. administration of *N. kaouthia* monovalent antivenom (NKAV) for 30 min following experimental *N. kaouthia* envenomation (2 mg/kg, i.m.) show satellite cells (yellow arrowheads) and mitochondria (green arrowheads). (**g**) H&E staining image and (**h**) TEM analysis of the rat diaphragm after i.v. administration of NKAV at 90 min following the administration of *N. kaouthia* (2 mg/kg, i.m.) show the satellite cells (yellow arrowheads), red blood cell aggregations (blue arrow), intramyocellular lipid droplets (black arrowheads) and the mitochondria (green arrowheads). ‘Lu’ indicates the lumen of blood vessels. ‘En’ indicates endothelial cell swelling. ‘Nu’ indicates the nucleus. ‘S’ indicates the nucleus of satellite cells.

The administration of saline (i.m.) did not affect the nucleus or mitochondria of the rat diaphragms. No histopathological change was observed (Figure 1a, Figure 2a and Figure 3a,b). The administration of *N. kaouthia* venom (2 mg/kg, i.m.) for 60 min caused a mild skeletal muscle fibre atrophy and red blood cell aggregation (Figure 1b). Myofibril rupture, mitochondrial swelling and the presence of zig-zagging of the Z-band were observed following venom administration for 90 min (Figure 1c and Figure 2b,c). Following the i.m. administration of *N. kaouthia* venom for 4 h, severe muscle fibre degeneration, red blood cell aggregation and a high degree of mitochondrial swelling and rupture including intramyocellular lipid droplets were detected (Figure 1d, Figure 2d,e and Figure 3c,d, Table 2).

### 3.3. The Effectiveness of NKAV on Histopathological Changes Following N. kaouthia Envenoming

Venom-induced pathological changes in the rat diaphragm were minimised by the intravenous administration of *N. kaouthia* antivenom (NKAV at the recommended therapeutic dose: 1 mL antivenom per 0.6 mg *N. kaouthia* venom) after the administration of *N. kaouthia* venom (2 mg/kg, i.m.) for 30 (Figure 3e,f) and 90 min. The administration of NKAV at 90 min after *N. kaouthia* envenoming (2 mg/kg, i.m.) partially decreased mitochondrial swelling of the diaphragm (Figure 3g,h). However, red blood cell aggregation and intramyocellular lipid droplets in the envenomed diaphragm were detected following NKAV administration (i.v.) at 90 min after envenoming (Figure 3g,h, Table 3).

### 3.4. Histopathological Examination of Neuromuscular Junction in Rat Diaphragm Following i.m. Administration of N. kaouthia Venom

**Figure 4:**
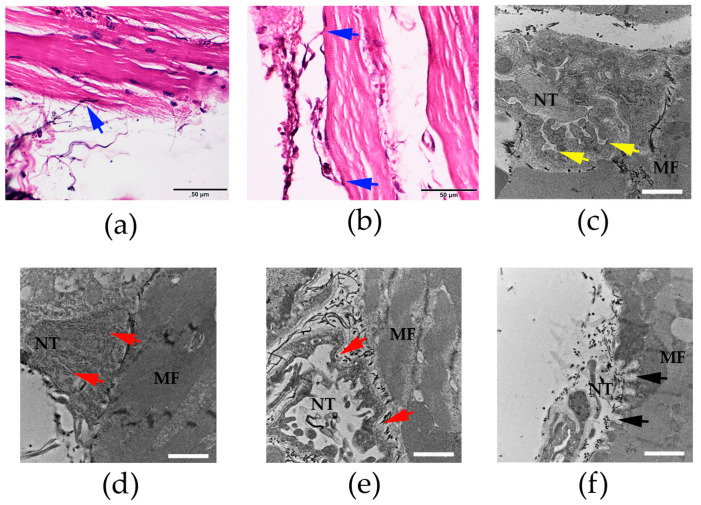
Morphological changes of the rat diaphragm (H&E staining; scale bar = 50 µm) after the i.m. administration of (**a**) saline (200 µL) and (**b**) *N. kaouthia* venom (2 mg/kg) showing the nerve terminal of nerve fibres (blue arrows). (**c**) Transmission electron microscopy image (TEM: scale bar = 2 µm) of the rat diaphragm following the administration of saline showing the synaptic folds of the neuromuscular junction (yellow arrows). (**d**) The administration of *N. kaouthia* venom (2 mg/kg: i.m.) for 90 min caused partial degeneration of the synaptic fold under TEM analysis (red arrows: scale bar = 2 µm). (**e**) TEM image of rat diaphragm shows severe degeneration and loss of the synaptic fold (red arrows) following i.m. administration of *N. kaouthia* venom (2 mg/kg) for 4 h. (**f**) The appearance of the synaptic fold (black arrows) in the rat diaphragm (TEM analysis: scale bar = 2 µm) after i.v. administration of NKAV at 30 min following the administration of *N. kaouthia* (2 mg/kg, i.m.). ‘NT’ indicates nerve terminal. ‘MF’ indicates muscle fibre.

The histopathological study of the rat diaphragm using H&E staining exhibited the phrenic nerve terminal attaching with the rat diaphragm muscle (Figure 4a,b). The administration of saline did not cause a marked histopathological change of the neuromuscular junction in the diaphragm (Figure 4a,c). The administration of *N. kaouthia* venom (2 mg/kg: i.m.) for 90 min (Figure 4d) caused the degeneration of synaptic folds. The loss of the synaptic fold was detected following the intramuscular administration of *N. kaouthia* venom for 4 h (Figure 4e). The administration of NKAV at 30 min prevented the degeneration of the synaptic fold following the administration of *N. kaouthia* venom (2 mg/kg, i.m.; Figure 4f).

### 3.5. Efficacy of Cobra Monovalent Antivenom in N. kaouthia Venom in Proinflammatory Cytokines in Diaphragm Tissues

**Figure 5:**
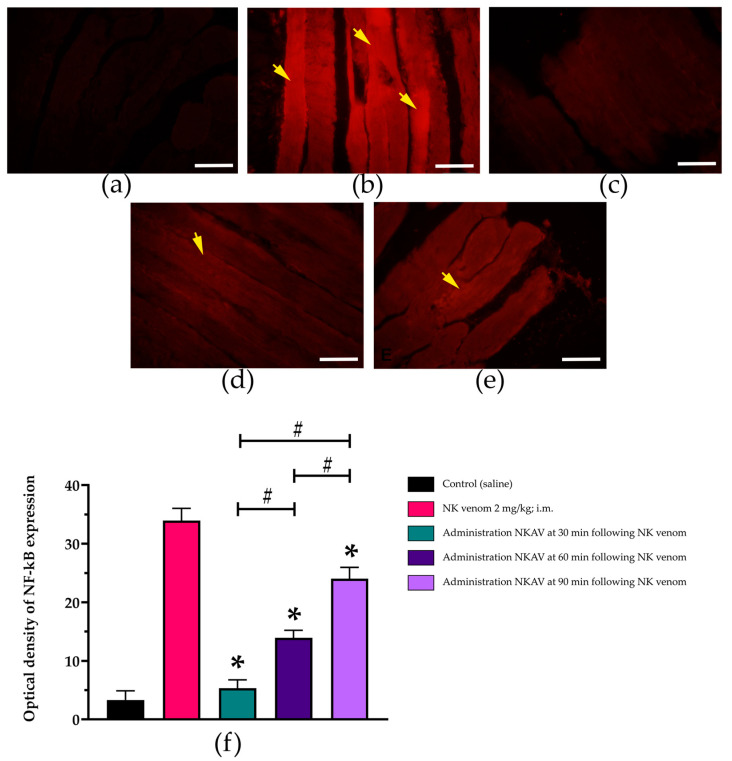
Immunofluorescence analysis (scale bar = 50 µm) of the stimulation of NF-kB in the rat diaphragm tissues following the administration of (**a**) saline, (**b**) *N. kaouthia* venom (NK venom 2 mg/kg, i.m.), (**c**) *N. kaouthia* antivenom (NKAV, i.v.) following *N. kaouthia* venom (2 mg/kg, i.m.) at 30 min, (**d**) *N. kaouthia* antivenom (NKAV, i.v.) following *N. kaouthia* venom (2 mg/kg, i.m.) at 60 min and (**e**) *N. kaouthia* antivenom (NKAV, i.v.) following *N. kaouthia* venom (2 mg/kg, i.m.) at 90 min. (**f**) A comparison of optical density of NF-kB expression. Data represent the mean ± SEM (* *p* < 0.0001 compared with the group of NK venom (2 mg/kg, i.m.): # *p* < 0.0001, there was a significant difference when comparing between the groups, one-way analysis of variance (ANOVA); *n* = 3–5). Yellow arrows indicate the increase in NF-kB expression.

**Figure 6:**
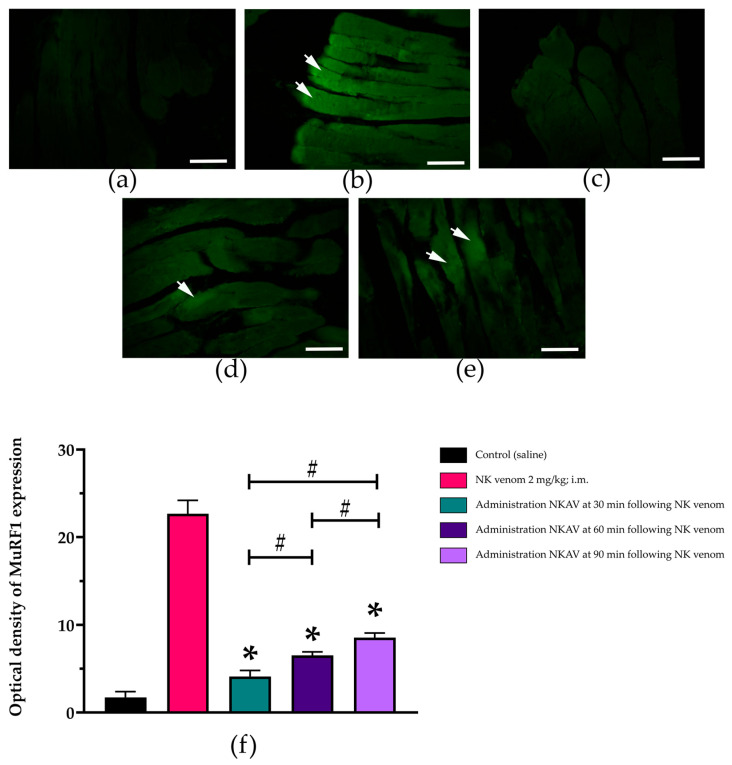
Immunofluorescence analysis (scale bar = 50 µm) of the stimulation of MuRF1 in the rat diaphragm tissues following the administration of (**a**) saline, (**b**) *N. kaouthia* venom (NK venom 2 mg/kg, i.m.), (**c**) NKAV (i.v.) following *N. kaouthia* venom (2 mg/kg, i.m.) at 30 min, (**d**) NKAV (i.v.) following *N. kaouthia* venom (2 mg/kg, i.m.) at 60 min and (**e**) NKAV (i.v.) following *N. kaouthia* venom (2 mg/kg, i.m.) at 90 min. (**f**) The comparison of optical density of MuRF1 expression. Data represent the mean ± SEM (* *p* < 0.0001 compared with NK venom (2 mg/kg, i.m.), # *p* < 0.0001, there was a significant difference when comparing between the groups, one-way ANOVA; *n* = 3–5). White arrows indicate the increase in MuRF1 expression.

The administration of saline did not cause visible nuclear factor-kappa B (NF-κB) or muscle-specific RING finger protein-1 (MuRF1) expression in the rat diaphragm (Figure 5a and Figure 6a).

The administration of *N. kaouthia* venom (2 mg/kg, i.m.) for 4 h significantly increased the optical density (OD) of NF-κB expression (*n* = 3–5; * *p*-value ≤ 0.0001; Figure 5b,f). The administration of *N. kaouthia* monovalent antivenom (NKAV) at 30 (Figure 4c), 60 (Figure 5d) and 90 min (Figure 5e) following experimental *N. kaouthia* envenoming caused a significant decrease in NF-κB expression. There were significant decreases in NF-κB expression between the time differences in the antivenom administration (*n* = 3–5; # *p*-value ≤ 0.0001; Figure 5f).

Administration of *N. kaouthia* venom (2 mg/kg, i.m.) also induced the higher expression of MuRF1 (*n* = 3–5; * *p*-value ≤ 0.0001; Figure 6b, one-way analysis of variance [ANOVA]) than that of saline and rats treated with *N. kaouthia* monovalent antivenom at 30 (Figure 6c), 60 (Figure 6d) and 90 min (Figure 6e) following the experimental *N. kaouthia* envenoming (*n* = 3–5; # *p*-value ≤ 0.0001; Figure 6f).

## 4. Discussion

*N. kaouthia* (monocled cobra) is an endemic cobra species in Southeast Asia. Severe neurotoxicity and death due to infection of the bite wound and respiratory failure have been reported following envenoming by *N. kaouthia* in Thailand [6]. Neurotoxic symptoms, such as bilateral ptosis, persistently dilated pupils, limb weakness, breathlessness, dysphonia and dysphagia, are clinically important in the diagnosis and treatment of *N. kaouthia* envenoming. These envenomed outcomes are due to the effect of pre- and postsynaptic neurotoxins causing a transmission blockade in nerve–muscle connectivity. Many previous studies have focused on the inhibitory effects of snake neurotoxins on neuromuscular transmission either on the effect on the presynaptic nerve terminal or that on the postsynaptic nicotinic acetylcholine receptors. Studies on the pathological changes involving the respiratory muscles following snakebite envenoming are limited. Here, we used an in vivo rat model to investigate pathological changes in the diaphragm following experimental *N. kaouthia* envenoming. In the current study, *N. kaouthia* venom doses of 0.5 and 1.0 mg/kg did not significantly cause marked pathological effects on the diaphragm following i.m. administration. Subsequently, a dose of 2 mg/kg was chosen. In addition, a previous study showed that the average *N. kaouthia* venom yield is between 0.17 and 1.90 g [16]. Therefore, the cobra venom dose of 2 mg/kg used in the current study is correlated to the real-world scenario.

Studies on *N. kaouthia* venom LD_50_ (the dose required to kill half the members of tested animals in a specific duration) have reported different ranges of LD_50_ between 0.148 and 0.614 mg/kg [17,18]. Indeed, the difference in the LD_50_ of snake venom has been found to be associated with the route of administration, including proteomic variations that make venoms neutralised at different efficacies by antivenoms [14]. In the current study, a venom dose of 2 mg/kg was selected to induce the acute toxicity of respiratory muscles, which is equivalent to 4xLD_50_.

Examining snake venom neurotoxicity and the neutralising effect of snake antivenom has mostly been performed in in vitro studies using isolated skeletal muscles—either the chick biventer cervicis nerve–muscle preparation [19] or the isolated rodent diaphragm [20]. Species differences in the interaction between snake toxins and the ACh receptor binding site have been reported [21,22]. Moreover, the chick biventer cervicis nerve–muscle preparation is simpler and more robust in experiments than the rodent muscle preparation. It can be used to distinguish between the actions of neuromuscular blocking agents that cause depolarisation (i.e., succinylcholine or carbachol) and those that do not [23]. It has been reported that *N. kaouthia* venom at high concentrations caused a direct effect on skeletal muscle, resulting in contractures, loss of tension following direct stimulation and loss of sensitivity to elevated potassium [20]. In addition, a comparative study between *N. kaouthia* and *N. sumatrana* venoms showed a decrease in the contractile response of chick muscles to potassium following cobra venom incubation, thus suggesting the presence of a myotoxic effect of both venoms [24]. Using an in vivo model to determine the pathological effect of snake venom on skeletal muscle has rarely been performed. Myotoxicity following *N. kaouthia* envenoming is rarely observed in clinical settings. A retrospective cohort study of cobra envenoming in Thailand reported that 21.4% of the cobra envenoming population had rhabdomyolysis [6].

To the best of our knowledge, this work is the first in vivo study to determine pathological changes in the diaphragm following experimental *N. kaouthia* envenoming. We found that the pathological changes in the rat diaphragm following i.m. administration of *N. kaouthia* venom were time-dependent. In addition, we recently reported the tissue damages following snakebite envenoming for 3 h, which also involved snake venom pharmacokinetics [25]. The characteristic of skeletal muscle cells following snake venom/toxin incubation is myonecrosis, which includes vacuolation, oedematous fibre, haemorrhage and myofilament hypercontraction [26]. In this study, administration of *N. kaouthia* venom for 4 h affected mitochondrial structure, i.e., swollen and degeneration of mitochondria. Moreover, the presence of zig-zagging of the Z-band of muscle fibre following *N. kaouthia* venom administration indicates the contraction of muscle fibre [27]. Venom toxins with tissue-damaging capabilities can be classified into two categories based on their effects on cells. The first category is cytotoxins, defined as toxins that are ‘truly’ cytotoxic by directly affecting the viability of cells [28], such as 3FTxs, PLA_2s_ and β-defensin-like toxins [29]. These cytotoxins cause local and systemic myonecrosis [30] and skeletal muscle contracture. The second category is extracellular matrix-degrading enzymes, such as snake venom metalloproteinases (SVMPs) and hyaluronidases, which damage cells through indirect mechanisms. A recent study reported the proteomic profile of *N. kaouthia* venom to comprise enzymatic proteins (i.e., PLA_2s_, SVMPs, LAAO, phosphodiesterase and nucleotidase) and non-enzymatic compounds (i.e., 3FTx, cysteine-rich secretory proteins, cobra venom factor, nerve growth factor and Ohanin-like proteins), which induce skeletal muscle damage through direct and indirect mechanisms, respectively [31]. Moreover, degeneration of the synaptic fold following i.m. administration of *N. kaouthia* venom was also detected. This finding suggests that the mechanism behind cobra-envenoming-induced neurotoxicity also involves morphological changes of the neuromuscular junction either presynaptic or postsynaptic membrane.

The administration of monospecific antivenom remains an effective treatment for cobra envenoming. The indication for antivenom administration includes any clinical signs and symptoms of skeletal muscle paralysis or weakness. In this study, the i.m. administration of a 2 mg/kg dose of *N. kaouthia* venom for 60 min induced the aggregation of red blood cells, while ruptured myofibril and severe muscle hypertrophy gradually developed at 90 min. The pathological changes in the rat diaphragm following monocled cobra envenoming were abolished by the intravenous administration of NKAV at the recommended therapeutic dose (i.e., 1 mL antivenom per 0.6 mg of *N. kaouthia* venom). However, the effectiveness of snake antivenom depends on the time of administration following snakebite envenoming. Delayed antivenom administration at 90 min did not prevent red blood cell aggregation or some lesions in the cobra-envenomed tissues. A previous study on an anaesthetised rat showed that prior administration of a high-dose antivenom had a protective effect on the pathological changes in the renal tissue and myotoxicity following Russell’s viper envenoming. Therefore, the early administration of antivenom following snakebite envenoming is recommended for maximum benefit to neutralise snake venom toxicities [32].

The immunofluorescence analysis showed that *N. kaouthia* venom envenoming caused an increase in the inflammatory protein expression in the rat diaphragm. We investigated the association between *N. kaouthia*-venom-induced tissue damage and the expression of inflammatory and muscle remodelling proteins called NF-κB and MuRF1, respectively. NF-κB is a transcription factor in B-cells that involves a number of signalling events [33]. NF-κB plays an important role in regulating gene expression in immune response, oncogenesis, proliferation, differentiation, apoptosis and angiogenesis [34]. In skeletal muscle, NF-κB appears to be a crucial pathway associated with the loss of muscle mass in various pathophysiological conditions. The activation of NF-κB in skeletal muscle cells causes the degradation of specific muscle proteins, induces inflammation and fibrosis and inhibits the healing process of myofibres after injury and atrophy [35]. Conversely, MuRF1 is an E3 ubiquitin ligase selectively expressed in cardiac and skeletal muscle tissues. MuRF1 plays an important role in the muscle remodelling circuit. The upregulation or expression of the MuRF1 gene correlates with skeletal muscle atrophy [36]. In this study, we demonstrated that the diaphragm from *N. kaouthia*-envenomed rats exhibited a significant increase in the OD of NF-κB and MuRF1 expression, suggesting inflammatory and skeletal muscle atrophy events, respectively. The administration of *N. kaouthia* monovalent antivenom significantly inhibited both NF-κB and MuRF1 expressions in the cobra-envenomed diaphragm. The early administration of antivenom at 30 min exhibited a higher inhibitory action on the expression of NF-κB and MuRF1 compared with the administration of antivenom at 60 and 90 min.

This study has some limitations. As the anaesthetised rats were entirely used for the determination of vital signs and other pathological effects following *N. kaouthia* envenoming, the results may be different when conscious rats are used. Pathological studies on the diaphragm following cobra venom administration at 30 min should be performed to determine the early stage following snakebite envenoming in the respiratory muscle. In this study, the i.m. administration of *N. kaouthia* at a 2 mg/kg dose caused undetectable blood pressure and heart rate in rats within 4 h. Therefore, a termination time-point at 4 h following venom administration was chosen for tissue collection in all tested animals, including antivenom-treated rats. An extended termination time after antivenom administration can provide more information on the effectiveness of antivenom.

## 5. Conclusions

The most significant effect of envenoming by cobras is neurotoxicity, causing progressive neuromuscular paralysis and leading to respiratory failure. In this study, we demonstrated that *N. kaouthia* envenoming causes histopathological changes in the respiratory muscles, specifically the diaphragm muscle. The immunofluorescence (i.e., NF-κB and MuRF1 expressions) confirmed the inflammation and injury of skeletal muscle, respectively. Further pharmacological and physiological studies may enable a better understanding and management of cobra envenoming. Early antivenom administration and respiratory support (i.e., endotracheal intubation with ventilator support) are essential for the clinical management of systemic *N. kaouthia* envenoming. Our data also indicate that the morphological anomalies observed in envenomed tissues may contribute to myotoxicity and respiratory failure in envenomed victims. Early monitoring of respiratory function and appropriate snake antivenom administration are required to prevent life-threatening outcomes.

## Figures and Tables

**Table 1:** Pathological characteristic and evaluation criteria of TEM analysis for degree of morphological changes in diaphragms.

Numerical Score	Description
0	No lesions
1	Mild multifocal necrosis
2	Intramyocellular lipid droplets and moderate multifocal necrosis such as mitochondria swelling, hypercontraction of muscle fibre
3	Several intramyocellular lipid droplets, severe myofiber necrosis with mitochondria swelling and degeneration and the presence of zig-zagging Z-band.

**Table 2:** Pathological score of the diaphragm following the administration of saline and *N. kaouthia* venom (2 mg/kg; i.m.) for 90 min and 4 h under TEM analysis (*n* = 3–5).

	Diaphragm (Scores)
	Skeletal Muscle Damage
Control (saline)	0
*N. kaouthia* venom (2 mg/kg; i.m.) for 90 min	2
*N. kaouthia* venom (2 mg/kg; i.m.) for 4 h	3

**Table 3:** Pathological score of the diaphragm under TEM analysis following the administration of saline, *N. kaouthia* venom (2 mg/kg; i.m.) for 4 h and *N. kaouthia* antivenom (NKAV) at 30 and 90 min following *N. kaouthia* venom (*n* = 3–5).

Group	Diaphragm (Scores)
	Skeletal Muscle Damage
Control (saline)	0
*N. kaouthia* envenoming (2 mg/kg; i.m.) for 4 h	3
Administration of NKAV at 30 min following *N. kaouthia* envenoming	1
Administration NKAV at 90 min following *N. kaouthia* envenoming	2

## Data Availability

The datasets used and/or analysed during the current study are available from the corresponding author (J.C.) upon reasonable request.
